# Machine learning-based risk predictive models for depression in patients with diabetes: a systematic review and meta-analysis

**DOI:** 10.3389/fendo.2026.1816661

**Published:** 2026-04-15

**Authors:** Xingxin Cai, Guiying Guo, Jun Zhou, Mengqi Han, Yuanyuan Cui, Zhenglin Chen

**Affiliations:** 1 School of Nursing, Shanxi University of Chinese Medicine, Jinzhong, Shanxi, China; 2 Department of Endocrinology, Shanxi Integrated Traditional Chinese and Western Medicine Hospital, Taiyuan, Shanxi, China

**Keywords:** depression, diabetes mellitus, machine learning, meta-analysis, predictive model, systematic review

## Abstract

**Background:**

Currently, numerous studies have employed machine learning (ML) methods to develop predictive models for depression risk in patients with diabetes mellitus (DM); however, the findings remain inconsistent. Therefore, this study aims to clarify the current state of research and emerging trends in this field by systematically evaluating the performance, strengths, and limitations of existing prediction models.

**Objective:**

This systematic review evaluates the performance and clinical applicability of ML-based depression risk prediction models for patients with DM, providing reliable evidence to assist healthcare professionals in selecting and optimizing more appropriate prediction models.

**Methods:**

We conducted a systematic search of clinical studies employing ML approaches to predict depression risk in patients with DM across the PubMed, Embase, Cochrane Library, and Web of Science databases, from their inception to January 2026. The primary performance metric for the models was the area under the receiver operating characteristic curve (AUC) along with its 95% confidence interval (95% CI). Two independent researchers screened the literature, extracted data, and used PROBAST-AI to assess the risk of bias and clinical applicability of the included studies. Pooled AUC was estimated using the Der Simonian and Laird random-effects model.

**Results:**

A total of 14 studies comprising 64 distinct ML models were included. All included studies were assessed as high risk of bias and high clinical applicability. A pooled analysis of the best-performing ML prediction models reported in each study showed a pooled AUC of 0.822 (95% CI, 0.789-0.858), indicating relatively good overall predictive performance. However, there was substantial heterogeneity among the studies (*I²* = 97.4%; *P* < 0.001). Subgroup analysis based on ML model types revealed the following pooled AUC values: 0.765 (95% CI 0.706-0.829) for traditional regression models, 0.789 (95% CI 0.747-0.834) for general machine learning models, and 0.802 (95% CI 0.769-0.836) for deep learning models. Notably, logistic regression (LR) (n = 10) was the most frequently employed ML method for developing depression risk prediction models in patients with DM. To evaluate model generalizability and avoid overfitting, the included studies adopted three validation strategies: 5-fold cross-validation yielded a pooled AUC of 0.913 (95% CI 0.781-1.067), 10-fold cross-validation yielded 0.819 (95% CI 0.781-0.858), and random split validation yielded 0.747 (95% CI 0.648-0.862). The most commonly used predictors in the included models were age, sex, and body mass index (BMI), which are readily available in clinical settings and strongly associated with depression risk.

**Conclusions:**

ML-based depression risk prediction models for patients with DM demonstrate overall satisfactory predictive performance. However, most existing studies had relatively small sample sizes and lacked external validation. Future research should prioritize refining study design and optimizing clinical data processing to improve the generalizability and stability of these models in clinical practice.

**Systematic Review Registration:**

https://www.crd.york.ac.uk/PROSPERO/view/CRD420251243343, identifier CRD420251243343.

## 1 Introduction

In recent years, the global prevalence of diabetes mellitus (DM) has been continuously increasing, rising from 537 million in 2021 to 589 million in 2024, and is projected to further increase to 853 million by 2050 (1). As a lifelong chronic metabolic disease, patients with DM require long-term adherence to self-management. Under the combined pressures of lifestyle modifications, physical distress, and concerns regarding complications, these patients are susceptible to developing depressive symptoms (2). Studies have demonstrated (3, 4) that at least one-third of patients with DM suffer from depression, and the risk of depression in patients with DM is twice as high as that in individuals without DM. More importantly, a clear bidirectional association exists between depression and DM: patients with depression are more likely to develop DM, while a diagnosis of DM increases the risk of developing depression (5). When both conditions coexist, they not only reduce patients’ quality of life and self-management capacity, leading to suboptimal glycemic control and elevated risks of macrovascular and microvascular complications, but also increase patient mortality (6, 7). Therefore, the early identification and active intervention of depressive states in patients with DM are of critical importance.

Currently, the widely utilized depression assessment tools in clinical practice, including the Patient Health Questionnaire-9 (PHQ-9), the Center for Epidemiologic Studies Depression Scale (CES-D), and the Hospital Anxiety and Depression Scale (HADS), primarily assess the current severity of depressive symptoms in patients with DM, and provide preliminary evidence for clinical diagnosis, disease stratification, and early intervention. However, these tools demonstrate limited generalizability and poor predictive capacity for the future risk of depressive episodes and the progressive trajectory of depression in patients with DM. Furthermore, their accuracy and flexibility are constrained by predetermined scoring algorithms and inherent linear assumptions (8, 9).

With the rapid advancement of artificial intelligence and big data technologies, the application of machine learning (ML) in the medical field has become increasingly widespread (10). Compared with traditional regression models, ML algorithms can automatically extract features and identify latent patterns from massive, complex multidimensional data, thereby enabling accurate risk prediction and clinical decision support (11). Presently, these algorithms have been increasingly applied in the field of depression risk prediction among patients with DM. However, owing to substantial heterogeneity across studies in dataset selection, model development strategies, and missing data imputation approaches, the performance and clinical applicability of different models cannot be readily compared.

This study aims to systematically evaluate existing ML-based depression risk prediction models for patients with DM, comprehensively summarize the model development methods and performance metrics, and compare the predictive performance of ML models with that of traditional algorithms using the area under the receiver operating characteristic curve (AUC) and 95% confidence interval (CI) as core indicators. This review further seeks to clarify the current research landscape and to provide targeted recommendations, offering scientific evidence to support clinical practitioners in developing and implementing depression risk prediction models for patients with DM.

## 2 Materials and methods

### 2.1 Study design

The protocol for this systematic review was published in the International Prospective Register of Systematic Reviews (PROSPERO) under the registered number CRD420251243343. This system evaluation is reported in accordance with the Preferred Reporting Items for Systematic Reviews and Meta-Analyses (PRISMA) checklist.

### 2.2 Literature inclusion and exclusion criteria

Inclusion criteria were as follows: ① Study population: patients diagnosed with DM (12); ② Study content: development of ML-based depression risk prediction models for patients with DM; ③ Outcome measure: the occurrence of depression as the predicted outcome; ④ Study designs including cohort studies, case-control studies, and cross-sectional studies. Exclusion criteria were as follows: ① Studies that failed to specify the ML algorithms used or only employed traditional regression models; ② Studies that merely analyzed influencing factors without developing a prediction model; ③ Studies not published in English; ④ Studies with unavailable full texts or from which valid information could not be extracted.

### 2.3 Literature search strategy

A systematic search was conducted across four databases: PubMed, Embase, Web of Science, and Cochrane Library. The screening process was conducted using a combination of subject headings and keywords, specifically “Diabetes Mellitus/diabetic/type 1 diabetes mellitus/type 2 diabetes mellitus,” “depression/depressive disease/depressive disorder,” “Forecasting/prediction/predictive model/risk prediction/risk score/risk assessment,” and “Machine Learning/Artificial Intelligence/Transfer Learning/Logistic regression/Decision trees/random forest/support vector machine.” Additionally, the references of the included literature were further traced to supplement the selection, ensuring comprehensiveness in the final set of publications. The search encompassed records from the inception of each database through January 2026. The search strategy is based on PubMed as an example (**Supplementary Figure 1**).

### 2.4 Literature screening and information extraction

This systematic review utilized EndNote 21 reference management software for deduplication. Two independent researchers screened the literature, extracted data, and cross-validated the extracted data. In cases of disagreement, they consulted a third party. For literature screening, we first reviewed the titles and abstracts; after excluding irrelevant studies, we further reviewed the full texts to determine inclusion. Data extraction followed the Cochrane guidelines and adhered to the Critical Appraisal and Data Extraction Checklist for the Systematic Evaluation of Predictive Models (CHARMS) (13). The extracted data included: first author, publication year, country, study type, study subject, sample source, sample size, assessment tools, predictive factors, methods for selecting predictive factors, modeling methods, validation methods, optimal algorithm, model checking methods, missing data handling methods, and AUC with its 95% CI.

### 2.5 Risk of bias assessment

Two independent researchers used PROBAST-AI, a tool designed to assess the methodological quality of individual prognostic or diagnostic multivariable prediction model studies, to assess the included studies (14). The assessment covered two dimensions: risk of bias and clinical applicability. Disagreements were resolved by consulting a third party. The risk of bias assessment covered four domains: study participants, predictors, outcomes, and data analysis (a total of 18 signal questions), using a three-level scoring system: “Yes”/”Probably Yes”, “No”/”Probably No”, or “Unclear”. The applicability assessment covered three domains: study participants, predictors, and outcomes, using a three-level evaluation: “High”, “Low”, and “Unclear”. If all domains were rated as low, the overall risk of bias was assessed as low; if at least one domain was judged to have a high risk of bias, the overall risk of bias was assessed as high; if at least one domain was judged as unclear and all other domains were rated as low, the overall risk of bias assessment was rated as unclear.

### 2.6 Statistical analysis

A meta-analysis was performed in Stata 18.0 to pool the AUC values and their 95% CIs from each included study. Subgroup analyses were conducted based on study design, model type, and other relevant factors. If the 95% CI or standard error (SE) of the AUC was not reported, we estimated the SE and 95% CI using the Hanley-McNeil formula (15–17). An AUC of 0.5 was considered to have no discriminatory ability, while an AUC of 1.0 was indicative of perfect discriminatory ability. The DerSimonian-Laird random-effects model was used to estimate the pooled AUC of the risk models (18), which can effectively account for between-study heterogeneity and aligns with the recommended methods for systematic reviews of diagnostic test accuracy and predictive modeling studies. Heterogeneity among the included studies was analyzed using the chi-square test (*α* = 0.1), and the magnitude of heterogeneity was quantitatively assessed using *I²* (19). Notably, *I²* ≤ 25% indicates low heterogeneity, 25% < *I²* ≤ 50% indicates moderate heterogeneity, and *I²* > 50% indicates high heterogeneity. We also estimated the approximate 95% prediction interval (PI) to describe the extent of heterogeneity among studies (20). We conducted sensitivity analyses by sequentially excluding individual studies to assess their impact on the pooled effect. Heterogeneity was assessed using either a fixed-effects or a random-effects model, and publication bias was assessed using Egger’s test; *p* > 0.05 indicated a low likelihood of publication bias.

## 3 Results

### 3.1 Literature screening process and results

**Figure 1:**
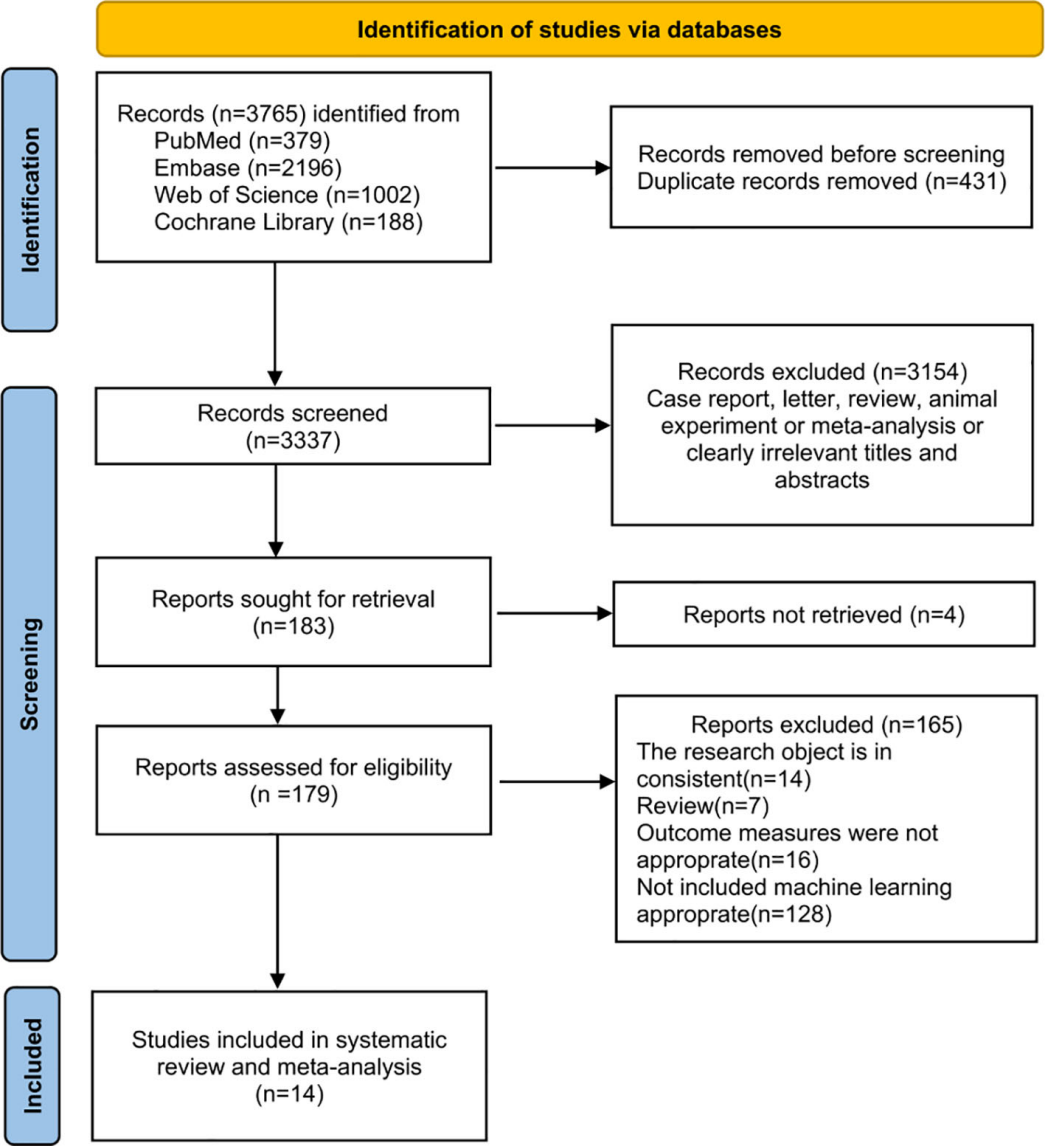
Study search and selection flowchart.

This study conducted a systematic search in multiple databases, initially identifying 3,765 studies. After the second screening based on the titles and abstracts, a total of 3154 studies were excluded. Additionally, 4 studies were excluded due to the inability to obtain the full texts. Eventually, 179 studies were subjected to full-text review. After screening, 14 studies met the inclusion criteria (21–34). A flowchart of the study search and selection process is detailed in **Figure 1**.

### 3.2 Basic characteristics of the included literature

A total of 14 studies were included in this systematic review, comprising 1 cohort study (21), 10 cross-sectional studies (22–31), and 3 case-control studies (32–34). Among them, 8 studies (21, 23, 25, 28–30, 33, 34) involved patients with type 2 diabetes mellitus (T2DM), while the remaining 6 studies (22, 24, 26, 27, 31, 32) focused on patients with DM in general. The overall sample size of the included studies ranged from 232 to 59,064 participants, which were utilized for model development and validation. Regarding depression assessment tools, 5 studies employed the PHQ-9 (21, 22, 24, 25, 29), and 3 utilized International Classification of Diseases (ICD)-10 codes (F32-F33, F40-F41) for depression diagnosis (32–34). The HADS, the Self-Rating Depression Scale (SDS), and the CES-D were each adopted in one study (26, 30, 31). Bataineh et al. (28) used the Diabetes Distress Scale (DDS) for assessment, whereas the remaining 2 studies (23, 27) did not specify the assessment tool used. The basic characteristics of the included studies were summarized in **Table 1**.

**Table 1:** The basic characteristics of the included studies.

Study	Country	Study type	Study subject	Sample source	Sample size	Assessment tools
Jin et al. (2019) (21)	United States	Cohort	T2DM	Diabetes–Depression Care-Management Adoption Trial	923	PHQ-9
Jin et al. (2015) (22)	United States	Cross	DM	Diabetes–Depression Care-Management Adoption Trial、Multifaceted Diabetes and Depression Program	1793	PHQ-9
Khalil et al. (2017) (23)	Australia	Cross	T2DM	Black Lion General Specialized Hospital	1320	clinical diagnosis
Lee et al. (2023) (24)	South Korea	Cross	DM	The Korean National Health and Nutrition Examination Survey	3007	PHQ-9
Yu et al. (2024) (25)	United States	Cross	T2DM	The USA National Health and Nutrition Examination Survey	4280	PHQ-9
Bourkhime et al. (2025) (26)	Morocco	Cross	DM	Hassan II University Hospital of Fez	243	HADS
Samsel et al. (2025) (27)	Canada	Cross	DM	Canadian Primary Care Sentinel Surveillance Network	7862	clinical diagnosis
Bataineh et al. (2025) (28)	United States	Cross	T2DM	Diabetes Distress Scale 17 dataset, United States	232	DDS
Su et al. (2025) (29)	China	Cross	T2DM	Taiwan Diabetes Registry	4188	PHQ-9
Duan et al. (2025) (30)	China	Cross	T2DM	The Quzhou Affiliated Hospital of Wenzhou Medical University	308	SDS
Deng et al. (2024) (31)	China	Cross	DM	China Health and Retirement Longitudinal Study	3443	CESD-10
Song et al. (2021) (32)	China	Case	DM	West China Hospital of Sichuan University	322	ICD-10 categories
Wei et al. (2024) (33)	China	Case	T2DM	Nanjing Health Information Platform、First Affiliated Hospital of Nanjing Medical University	18519	ICD-10 categories
Wei et al. (2025) (34)	China	Case	T2DM	Nanjing Health Information Center	59064	ICD-10 categories

Cross, cross-sectional study; Cohort, Cohort Study; Case, Case-Control Study; T2DM, Type 2 Diabetes Mellitus; DM, Diabetes Mellitus; PHQ-9, Patient Health Questionnaire-9; HADS, Hospital Anxiety and Depression Scale; DDS, Diabetes Distress Scale; SDS, Self-rating Depression Scale; CESD-10, Center for Epidemiologic Studies Depression Scale-10; ICD-10 categories, International Classification of Diseases-10 codes.

### 3.3 Types of machine learning algorithms and model performance

A total of 14 studies were included in this systematic review, collectively employing 19 distinct ML algorithms to develop 64 predictive models, among which logistic regression (LR) was the most frequently used, with 10 applications. It is worth noting that among the studies using LR, 4 studies (21, 25, 30, 31) only adopted this algorithm to construct the final predictive models, but all completed feature selection by combining machine learning techniques such as LASSO regression in the stage of predictor screening, which were classified as machine learning-assisted hybrid models. Pure traditional logistic regression models had been strictly excluded in accordance with the inclusion criteria. Regarding validation strategies, only 2 studies (33, 34) employed external validation; the remaining 12 used internal validation, predominantly using k-fold cross-validation (5-fold: n = 3; 10-fold: n = 8). All included studies reported the successful identification of an optimal model based on prespecified performance criteria. AUC values were reported in 12 of the 14 studies, with the exceptions of Khalil et al. (23) and Bourkhime et al. (26). Among the reported AUCs, the range was 0.700-0.980. Predictor selection varied across the included studies; however, sex (n = 7), age (n = 6), and body mass index (BMI) (n = 5) were consistently identified as the most frequently selected and statistically significant predictors. The establishment of the prediction model is shown in **Table 2**. The performance of the prediction model and the prediction factors are presented in **Table 3**.

**Table 2:** The establishment of the prediction model.

Study	ML algorithms	Validation methods	Optimal algorithm	Model checking methods	Missing data handling
Jin et al. (2019) (21)	LR	Internal validation	LR	10-fold cross-validation	complete case analysis
Jin et al. (2015) (22)	LR、SVM、RF、MLP	Internal validation	LR	10-fold cross-validation	—
Khalil et al. (2017) (23)	SVM、K-MEAN、F-CMEAN、PNN	Internal validation	SVM	Random sampling	—
Lee et al. (2023) (24)	RF、KNN、SVM、LightGBM、XGBoost、 AdaBoost	Internal validation	SVM	5-fold cross-validation	Multiple imputations
Yu et al. (2024) (25)	LR	Internal validation	LR	10-fold cross-validation	Multiple imputations
Bourkhime et al. (2025) (26)	LR、KNN、DT、RF、AdaBoost、SVM、 XGBoost、GatBoost	Internal validation	RF	5-fold cross-validation	mean imputation
Samsel et al. (2025) (27)	LR、NB、RF、AdaBoost、XGBoost、ANN	Internal validation	XGBoost	Random sampling	Multiple imputations
Bataineh et al. (2025) (28)	LR、RF、SVM、NN、XGBoost-SHAP、 XGBoost、GatBoost、Stacking	Internal validation	XGBoost- SHAP	5-fold cross-validation	Multiple imputations
Su et al. (2025) (29)	LR、RF、DT	Internal validation	LR	Random sampling	Multiple imputations
Duan et al. (2025) (30)	LR	Internal validation	LR	10-fold cross-validation	—
Deng et al. (2024) (31)	LR	Internal validation	LR	10-fold cross-validation	Multiple imputations
Song et al. (2021) (32)	SVM	Internal validation	SVM	10-fold cross-validation	—
Wei et al. (2024) (33)	LR、RF、XGBoost、DAP	Internal validation+ external validation	DAP	10-fold cross-validation	mean imputation
Wei et al. (2025) (34)	REDAPM、RETAIN、GRU、LSTM、HCET、 BRLTM	Internal validation+ external validation	REDAPM	10-fold cross-validation	Multiple imputations

LR, Logistic Regression; SVM, Support Vector Machine; RF, Random Forest; MLP, Multilayer Perceptron; K-MEAN, K-Means Clustering; F-CMEAN, Fuzzy C-Means Clustering; PNN, Probabilistic Neural Network; KNN, K-Nearest Neighbors; LightGBM, Light Gradient Boosting Machine; XGBoost, Extreme Gradient Boosting; AdaBoost, Adaptive Boosting; DT, Decision Tree; CatBoost, Categorical Boosting; NB, Naive Bayes; ANN, Artificial Neural Network; XGBoost-SHAP, XGBoost with SHapley Additive exPlanations; Stacking, Stacked Generalization; DAP, Deep Attention Pooling; REDAPM, Risk Estimation Using Deep Attention and Pooling Model; RETAIN, REverse Time AttentIoN model; GRU, Gated Recurrent Unit; LSTM, Long Short-Term Memory; HCET, Hierarchical Context Encoding Transformer; BRLTM, Bayesian Recurrent Latent Time Model.

**Table 3:** The performance of the prediction model and the prediction factors.

Study	Significant predictors	Variable selection	AUC (95%CI)
Jin et al. (2019) (21)	PHQ-2 score、SDS score、PBD、DSS score、DEB	LASSO、 Multivariate analysis	0.830 [0.779;0.881]
Jin et al. (2015) (22)	Female、Toobert diabetes self-care score、Total number of diabetes complications、 Previous diagnosis of major depressive disorder、Number of ICD-9 diagnoses in past 6 months、 Chronic pain、Self-rated health status	LASSO、 Multivariate analysis	0.810 [0.783;0.838]
Khalil et al. (2017) (23)	Sex、age、Residence、Marital status、Educational status、Monthly income、 Waist circumference、Diabetes treatment、BMI、Number of co-morbidity、Pill burden、 Number of diabetic complication、Physical disability、Negative life event、Poor social support、Fear of diabetic complication、Health care cost、Doing physical activity	—	—
Lee et al. (2023) (24)	Subjective health status、BP1、mh stress、ainc、HE TG、LQ4_00、EuroQoL、LQ1_sb、 Physical discomfort in the last 2 weeks、BM7	Boruta algorithm	0.835 [0.730;0.901]
Yu et al. (2024) (25)	Age、Sex、PIR、BMI、Education attainment、Smoking、LDL-C、Sleep duration、 Sleep disorder	LASSO、 Multivariate analysis	0.752 [0.711;0.793]
Bourkhime et al. (2025) (26)	Age、HBA1C、Therapeutic protocol、BMI、Marital status	Extra Trees Classifier	—
Samsel et al. (2025) (27)	Sex、Age、Osteoarthritis、BMI、HBA1C	SHAP	0.700 [0.661;0.739]
Bataineh et al. (2025) (28)	Age、Sex、Smoking、Food Type、Marital Status、Disease Since、FBS、Emotional Burden、 Physician Related Distress、Regimen Related Distress、Interpersonal Distress	Multivariate analysis、 SHAP	0.980 [0.968;0.992]
Su et al. (2025) (29)	Quality of Life、Bloating、Autoimmune disease	Multivariate analysis	0.810 [0.723;0.897]
Duan et al. (2025) (30)	Sex、BMI、LDL-C、CIRS score	LASSO、 Multivariate analysis	0.710 [0.651;0.769]
Deng et al. (2024) (31)	Sex、Permanent address、Self-perceived health status、Lung disease、Arthritis、 Memory disorder、Life satisfaction、Social Activities、Cognitive Function score、ADL score	LASSO、 Multivariate analysis	0.812 [0.786;0.839]
Song et al. (2021) (32)	Magnesium、Cholesterol、AST/ALT、Percentage of monocytes、Bilirubin indirect、 Triglyceride、LDH、Diastolic blood pressure	Recursive Feature Elimination	0.720 [0.558;0.882]
Wei et al. (2024) (33)	Postherpetic neuralgia diagnosis、Finasteride、Burn and corrosion, body region unspecified、 Acute myocardial infarction、Subarachnoid hemorrhage、Recombinant lysine-protein zinc insulin、Recombinant human insulin zinc、Nonorganic sleep disorders、Telmisartan、 Other inflammatory liver diseases	Integrated Gradients	0.910 [0.894;0.926]
Wei et al. (2025) (34)	Flupentixol、Mental disorder、Sleep disorders、Hydrocortisone、 Nimesulide、 SchizophreniaHeadache syndromes、Tazobactam、Alprazolam、Psoriasis	Recursive Feature Elimination、Integrated Gradients	0.903 [0.901;0.905]

PHQ-2, Patient Health Questionnaire-2; SDS, Self-rating Depression Scale; PBD, Probable Bipolar Disorder; DSS, Diabetes Symptom Scale; DEB, Diabetes Emotional Burden; BP1, Blood Pressure 1; HE TG, High Triglycerides; LQ4_00, Lifestyle Questionnaire 4_00; LQ1_sb, Lifestyle Questionnaire 1_sb; PIR, Predicted Insulin Resistance; BMI, Body Mass Index; LDL-C, Low-Density Lipoprotein Cholesterol; HbA1c, Glycated Hemoglobin A1c; FBS, Fasting Blood Sugar; CIRS, Cumulative Illness Rating Scale; ADL, Activities of Daily Living; AST, Aspartate Aminotransferase; ALT, Alanine Aminotransferase; LDH, Lactate Dehydrogenase.

### 3.4 Literature bias risk and applicability assessment

According to the PROBAST-AI evaluation criteria, all 14 included studies were assessed as having a high risk of bias. In the study participants’ domain, 13 studies (22–34) were rated as having a high risk of bias, primarily because the study data were derived from retrospective or cross-sectional studies. In the predictors domain, 13 studies (22–34) were rated as having a high risk of bias; among these, 7 studies (22, 24, 25, 27, 29, 31, 33) used multicenter data, with variations in the collection processes and measurement criteria of predictors, which ultimately led to a high risk of bias. 13 studies (22–34) were rated as having a high risk of bias because they were retrospective, and the outcome events may have occurred prior to the measurement of predictors, thereby introducing bias. In the outcomes domain, 3 studies (21, 23, 28) were rated as having a high risk of bias; among these, 1 study (23) failed to provide a detailed definition of outcome events, 1 study (28) had issues with the determination of outcome events, and the other 1 studies (21) exhibited a certain degree of bias in the results since information on early predictors of depression was already available when determining the outcomes. In the data analysis domain, 11 studies (21–23, 26–30, 32–34) were rated as having a high risk of bias, primarily due to an Events Per Variable (EPV) < 10 (23, 27), converting some continuous variables to categorical variables (22, 23, 26, 33, 34), failing to report information on missing data imputation methods (21–23, 30, 32), failing to use appropriate methods for predictor selection (22, 23, 28), and failing to report or evaluate discrimination and calibration (22, 23, 26–29, 32–34). Regarding overall clinical applicability, the 14 included studies demonstrated good applicability across study participants, predictors, and outcomes, and the overall clinical applicability was assessed as high. The comprehensive results of the overall risk of bias and clinical applicability assessment of the included studies were summarized in **Supplementary Table 1**.

### 3.5 Meta-analysis

**Figure 2:**
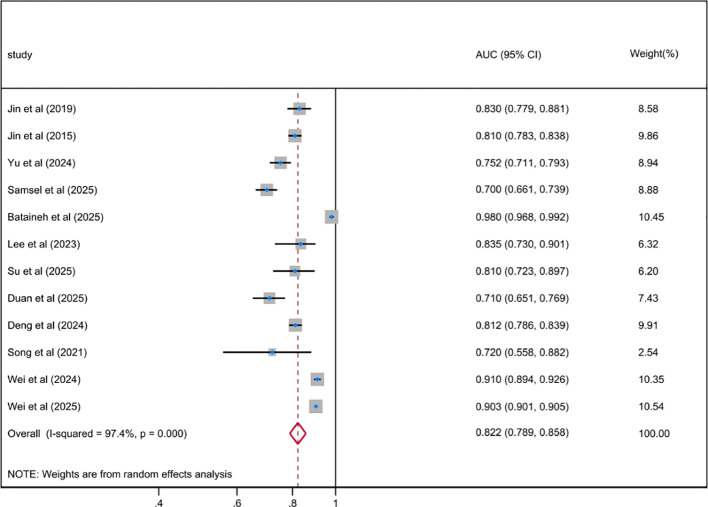
Random effects forest plot of the AUC of the best ML-based prediction model for depression risk in DM.

The pooled AUC of the best ML-based depression risk prediction models for patients with DM included in this systematic review was 0.822 (95% CI 0.789-0.858), with substantial heterogeneity among the included studies (*I²* = 97.4%; *P* < 0.001), as shown in **Figure 2**. After further meta-regression analyses, the results demonstrated that heterogeneity among the included studies remained high with no significant reduction. Egger’s value of -3.81 (*p* = 0.028) suggests that there was no significant publication bias; however, the potential for bias needs to be considered with caution, as shown in **Supplementary Figure 2**. The sensitivity analysis results demonstrated that the pooled AUC and 95% CI did not change significantly before and after sequentially excluding individual studies, with a fluctuation range of 0.805 (95% CI 0.763-0.848) to 0.837 (95% CI 0.800-0.873), indicating good stability of the pooled results, as shown in **Supplementary Figure 3**.

Owing to substantial heterogeneity, subgroup analyses were conducted based on study design type, region, modeling method, sample size, and overfitting mitigation approach. The results demonstrated that the pooled effect across subgroups did not change significantly, and the heterogeneity among subgroups did not decrease significantly. Subgroup analysis by study design type revealed that the pooled AUC for cross-sectional studies was 0.798 (95% CI 0.717-0.888), with substantial heterogeneity (*I²* = 98.3%; *P* < 0.001); the pooled AUC for case-control studies was 0.905 (95% CI 0.890-0.919). Subgroup analysis by region demonstrated that the pooled AUC for the Asian region (China, South Korea) was 0.837 (95% CI 0.801-0.875; *I²* = 92.8%; *P* < 0.001), while that for the non-Asian region (the United States, Australia, Canada, Morocco) was 0.810 (95% CI 0.703-0.933; *I²* = 98.7%; *P* < 0.001). The results of the meta-analysis were shown in **Supplementary Figures 4**, **5**.

**Figure 3:**
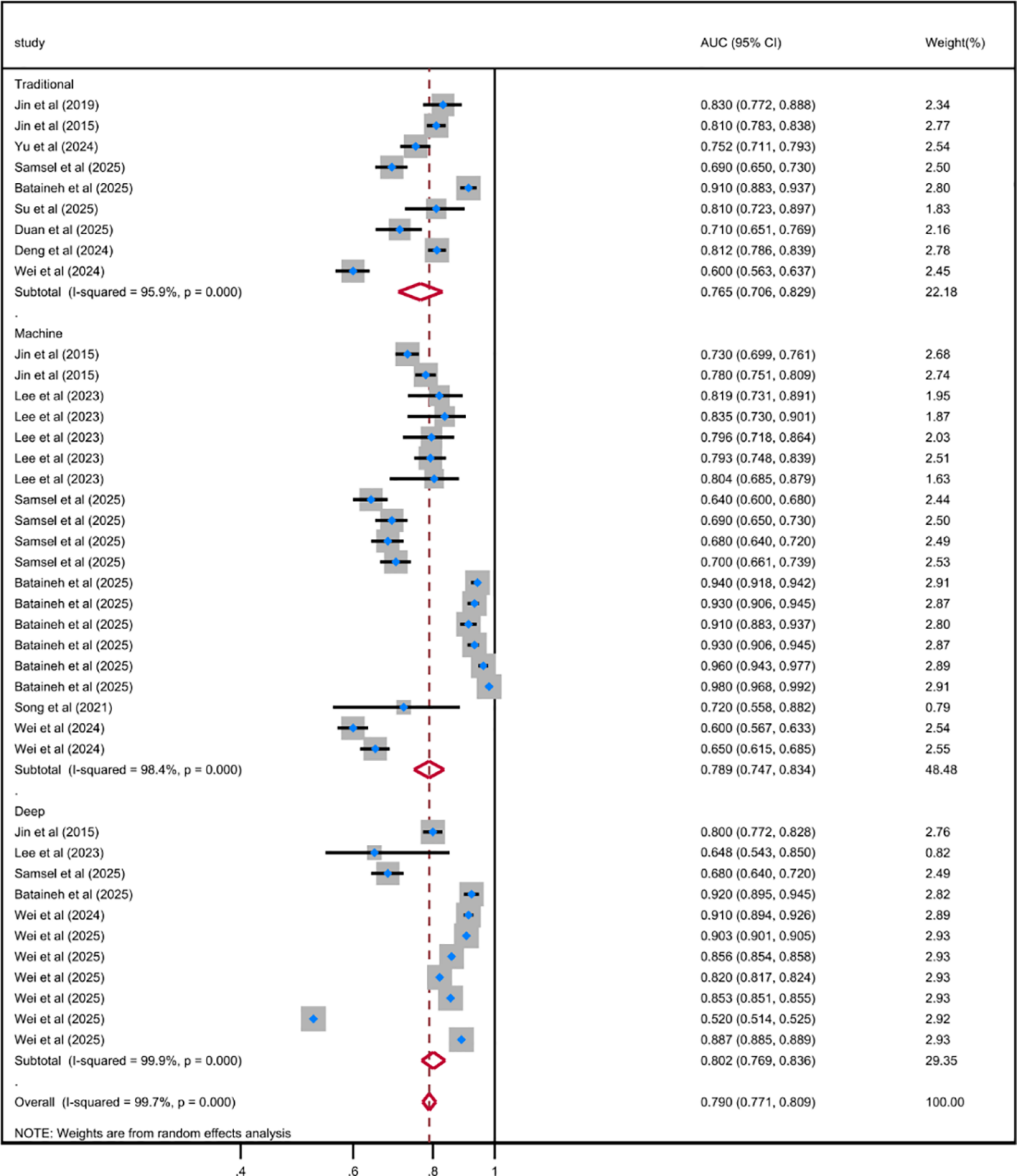
Random effects forest plot of AUC in model type subgroup for predicting depression risk in DM using ML.

**Figure 4:**
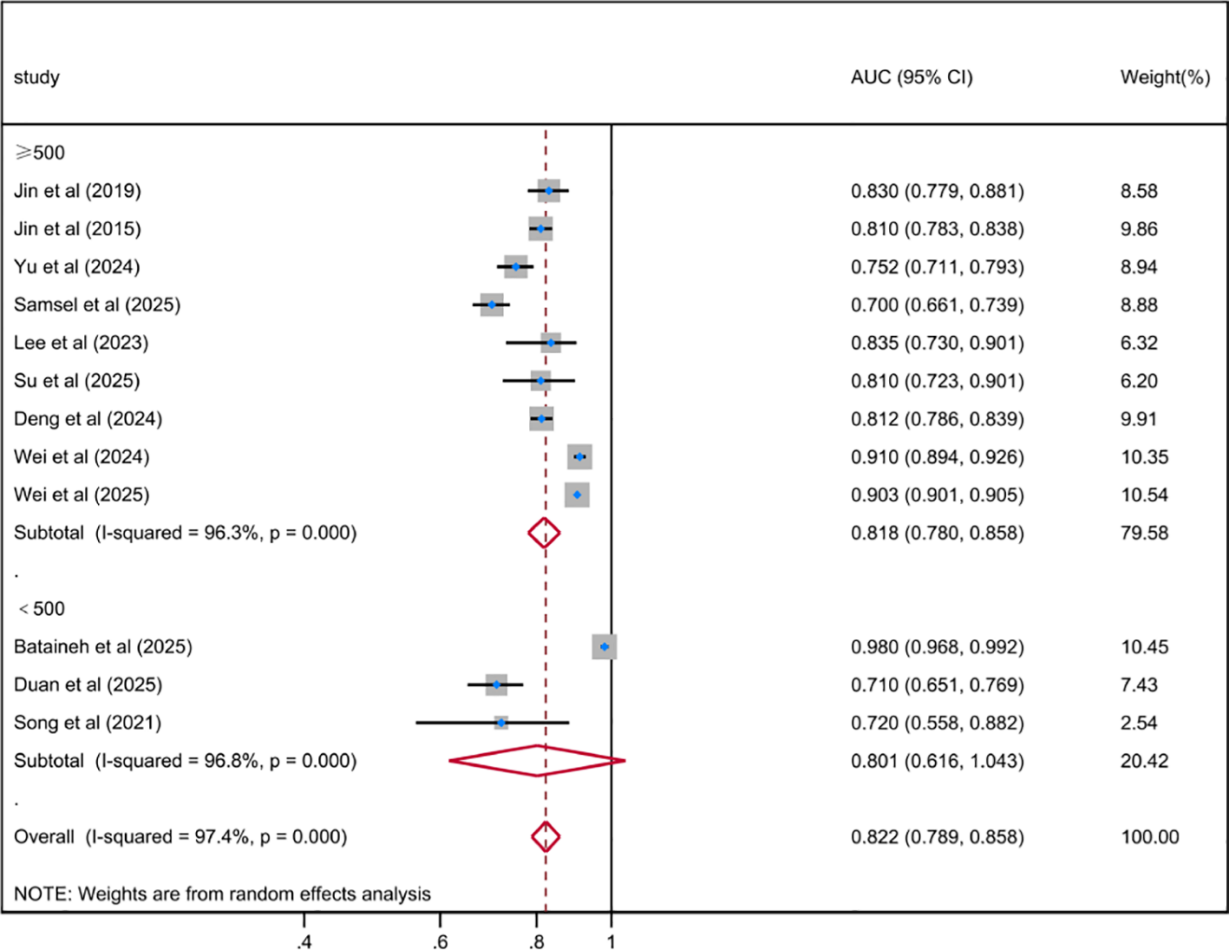
Random effects forest plot of AUC in sample size subgroup for predicting depression risk in DM using ML.

**Figure 5:**
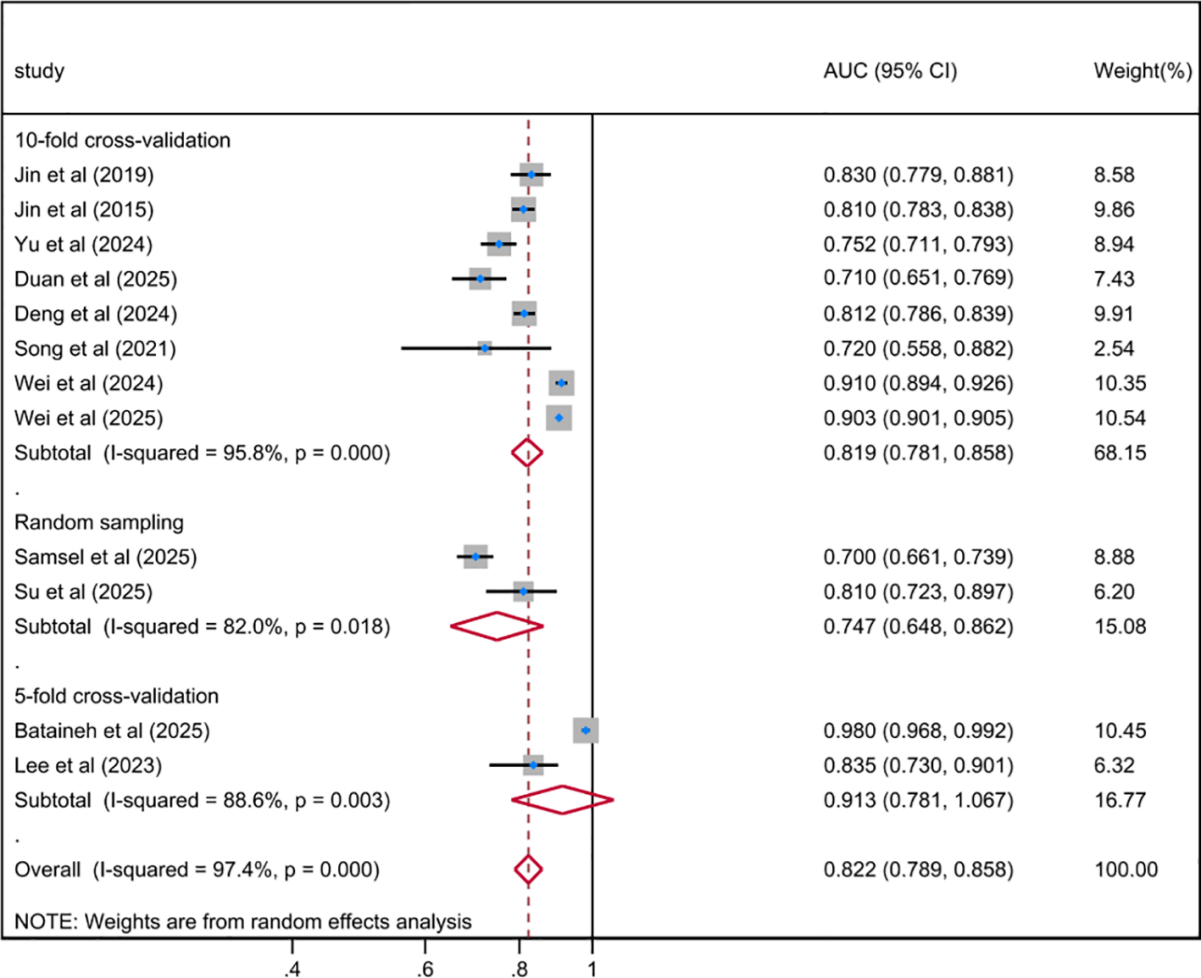
Random effects forest plot of AUC in fitting processing subgroup for predicting depression risk in DM using ML.

Based on the development history of ML, the algorithms included in the included studies were categorized into three types: traditional regression ML models (n = 9), general ML models (n = 20), and deep learning models (n = 11). The results indicated that the pooled AUC for traditional regression ML models was 0.765 (95% CI 0.706-0.829), with substantial heterogeneity (*I²* = 95.9%, *P* < 0.001); the pooled AUC for general ML models was 0.789 (95% CI 0.747-0.834), with even higher heterogeneity (*I²* = 98.4%, *P* < 0.001); compared with the first two types of models, the pooled AUC for deep learning models was 0.802 (95% CI 0.769-0.836); although it exhibited the highest level of heterogeneity (*I²* = 99.9%, *P* < 0.001), its overall predictive performance was still superior to that of traditional regression ML models and general ML models. The subgroup analysis based on sample size showed that the pooled AUC value of the model with a sample size of ≥ 500 cases was 0.818 (95% CI 0.780-0.858; *I^2^* = 96.3%; *P* < 0.001), while the pooled AUC value of the model with a sample size of < 500 cases was 0.801 (95% CI 0.616-1.043; *I^2^* = 96.8%; *P* < 0.001). Further subgroup analysis by the number of cross-validation folds demonstrated that the pooled AUC for 5-fold cross-validation was 0.913 (95% CI 0.781-1.067), and that for 10-fold cross-validation was 0.819 (95% CI 0.781-0.858); both cross-validation methods exhibited significant heterogeneity, indicating that methodological differences among the included studies may have influenced the consistency of predictive performance estimates. The results of the meta-analysis were shown in **Figures 3**–**5**. The results of the subgroup analysis were summarized in **Table 4**.

**Table 4:** The results of the subgroup analysis.

Subgroup	Number of prediction models	Heterogeneity test results		Meta-analysis results
		I^2^ value	P value	AUC(95%CI)
Study type				
Cross-sectional	8	98.3%	0.000	0.798(0.717-0.888)
Case-control	3	55.5%	0.106	0.905(0.890-0.919)
Country				
Non-Asian	5	98.7%	0.000	0.810(0.703-0.933)
Asian	7	92.8%	0.000	0.837(0.801-0.875)
Modeling methods				
Traditional	9	95.9%	0.000	0.765(0.706-0.829)
Machine	20	98.4%	0.000	0.789(0.747-0.834)
Deep	11	99.9%	0.000	0.802(0.769-0.836)
Sample size				
≥500	9	96.3%	0.000	0.818(0.780-0.858)
<500	3	96.8%	0.000	0.801(0.616-1.043)
Fitting processing				
Random sampling	2	82.0%	0.018	0.747(0.648-0.862)
10-fold cross-validation	8	95.8%	0.000	0.819(0.781-0.858)
5-fold cross-validation	2	88.6%	0.003	0.913(0.781-1.067)

## 4 Discussion

### 4.1 Performance of depression risk prediction models for DM patients based on ML algorithms

This systematic review synthesizes 14 studies that use ML to predict depression in patients with DM and compares their modeling approaches, predictor selection methods, and predictive performance. The pooled AUC of the included models was 0.822 (95% CI 0.789-0.858), indicating satisfactory discriminatory ability to effectively differentiate between high- and low-risk populations (35). However, there was considerable heterogeneity among the included studies (*I²* = 97.4%). Subgroup analyses based on various potential factors still revealed significant heterogeneity, suggesting that model performance varies across study populations, predictor sets, and modeling strategies. This is consistent with the real variability in clinical prediction modeling and calls for further exploration of its sources of heterogeneity.

It is hypothesized that differences in study design may be a key contributor to the observed heterogeneity. Subgroup analysis showed that models derived from case-control studies performed better. The reason for this is that case-control studies typically select cases with clear and typical diagnoses and corresponding non-diseased control groups for comparison, with a significant difference in outcome status between the two groups. This design feature makes it easier for the model to identify effective distinguishing factors, thereby enhancing its discriminative ability (36). Furthermore, geographical variation is also likely a major driver of heterogeneity. The included studies encompassed 6 countries, which were further categorized into Asian and non-Asian regions. Inherent differences in healthcare resource allocation, cultural attitudes, lifestyles, and socioeconomic conditions across these regions may influence model performance by altering exposure definitions, outcome assessment criteria, and the control of confounding variables (37). Subgroup analyses revealed that ML-based depression prediction models for patients with DM performed better overall in Asian populations compared to non-Asian populations. This may be attributed to smaller differences in genetic background and lifestyle among Chinese and Korean populations, leading to more homogeneous distributions of confounding factors and enabling models to identify determinants of depression risk more accurately.

ML methodologies hold the potential to achieve higher predictive accuracy by integrating diverse types of information, including conventional risk factors, genetic data, and clinical variables (38). However, it should be noted that the studies included in this review did not show any statistically significant differences in the predictive efficacy of different ML models. The predictive performance of deep learning models was comparable to that of traditional regression models and general machine learning models. Deep learning excels at automatically extracting high-dimensional predictors, uncovering complex nonlinear relationships and latent interaction effects from multi-source patient data that are often undetectable by traditional models (39). Yet, this advantage has not been fully reflected in the included studies. In contrast, traditional predictive models offer greater interpretability and tend to perform more robustly on small-to-medium-sized datasets, helping to mitigate overfitting (40). Nevertheless, ML methods are relatively poorly interpretable, require large amounts of data for training, and are prone to overfitting (41). Adequate sample size ensures model performance and avoids problems such as overfitting, yet an excessively large sample size does not further improve model performance (42). Subgroup analysis in the present study revealed that models with a sample size≥500 had better discrimination than those with a sample size <500, suggesting that appropriately expanding the sample size improves predictive performance when constructing risk prediction models. The method of dataset partitioning also influenced model performance; models employing 5-fold cross-validation showed significantly higher discriminatory ability than those using 10-fold cross-validation. Given that most included studies utilized small to moderate sample sizes, 5-fold cross-validation may better preserve data distribution and heterogeneity, thereby enhancing model discriminative power.

### 4.2 Bias risk analysis of depression risk prediction models for DM patients based on ML algorithms

In the assessment of 14 ML-based depression risk prediction models for patients with DM, most included studies demonstrated high predictive performance, with AUC values generally exceeding 0.80, and some studies even achieved values above 0.90. However, according to the PROBAST-AI criteria, all these included models were assessed as having a high risk of bias, primarily due to the following reasons (1): risk of bias in data sources (2); ambiguous outcome definitions (3); insufficient sample size (4); inappropriate handling of predictors (5); flawed approaches to managing missing data; and (6) lack of comprehensive performance evaluation for the prediction models.

Regarding study subjects, most studies (22–31) used a cross-sectional design, which may introduce bias because the outcome events had already occurred at the time of predictor assessment. Moreover, such studies are often conducted among a specific population at a single time point, potentially leading to selection bias and limiting the external validity of the prediction model. When retrospective studies were used for model development and validation (32–34), although data accessibility was enhanced, the included predictors might not be comprehensive, and missing data issues could further introduce bias into the results. It is recommended that future model development adopt prospective longitudinal cohort designs to mitigate such biases, with adequate follow-up duration to ensure the development and validation of models targeting incident depression risk. Regarding sample size, the PROBAST-AI framework suggests that, to minimize parameter estimation bias and avoid overfitting during model development, the number of positive outcome events should be at least 20 times the number of predictors—i.e., EPV ≥ 20. For model validation studies, a minimum of 100 subjects with outcome events is recommended (43). Two included studies (23, 27) failed to meet these sample size requirements during model development or validation, thereby increasing the risk of including irrelevant predictors or omitting significant ones. Concerning the treatment of predictors, 5 included studies (22, 23, 26, 33, 34) converted continuous predictors into categorical variables for statistical analysis. Such an approach often leads to substantial information loss, particularly for inherently continuous variables like age and BMI, as it eliminates fine-grained gradient information, thereby diminishing the model’s predictive power (44, 45). With respect to missing data imputation, 2 included studies (26, 33) employed mean imputation, 7 included studies (24, 25, 27–29, 31, 34) applied multiple imputation methods appropriately, while the remaining 5 included studies (21–23, 30, 32) either failed to explicitly report their missing data imputation strategy or utilized complete-case analysis. Inadequate handling of missing data may introduce systematic bias, compromising the accurate assessment of associations between predictors and outcomes, and ultimately impairing model performance (46). In terms of model performance evaluation, 9 included studies (22, 23, 26–29, 32–34) did not conduct calibration assessments, resulting in an incomplete evaluation of model effectiveness and potential risks of overfitting or underfitting (14). To ensure adequate generalizability, prediction models should undergo both internal and external validation before implementation to mitigate overfitting. In the present systematic review, only 2 included studies (33, 34) performed both internal and external validation, indicating a low rate of model validation and an elevated risk of bias.

### 4.3 Analysis of the common predictors in depression risk prediction models for DM patients based on ML algorithms

In clinical practice, clinical practitioners should not only place greater emphasis on the diagnosis of depression and anxiety but also understand the various influencing factors of depression in patients with DM. This enables the efficient identification and screening of patients with DM who exhibit adverse emotional states, thereby facilitating timely diagnosis and treatment, improving glycemic control, and reducing complications. The predictors included in the prediction models across the included studies reviewed herein are not entirely consistent but primarily encompass demographic data, disease-related risk factors, vital signs, and laboratory examinations. Notably, factors such as sex, age, BMI, marital status, treatment modality, and depression-related indicators have been repeatedly reported in multiple included studies.

Among the included studies in this systematic review, sex and age are fundamental predictors. Regarding sex, 7 included studies (22, 23, 25, 27, 28, 30, 31) consistently support its role as a significant predictive factor for depression risk in patients with DM. The study by Jin et al. (22) demonstrated that the probability of female patients with DM being predicted as depressed is 2.35 times that of males. This disparity may be attributed to factors such as greater psychological vulnerability, lower stress resilience, emotional susceptibility, and heightened physiological and psychological sensitivity to adverse life events among females (47, 48). In terms of age, six included studies (23, 25–28, 34) identified it as a predictor of depression risk. Both Yu et al. (25) and Sam et al. (27) demonstrated that older age serves as a protective factor against depression in patients with DM. In contrast, younger and middle-aged patients exhibit a higher risk of depression onset. This may be explained by the fact that young and middle-aged individuals are often at a critical stage of career advancement, bear greater familial and social responsibilities, and possess relatively lower psychological resilience compared to older adults, making them more prone to depressive symptoms under cumulative stress. However, the study by Wei et al. (34) had a different focus, revealing that the 40-80-year age group demonstrated superior predictive performance in the depression risk model. Such discrepancies may be related to variations in dataset characteristics and predictor selection across the included studies, necessitating further validation.

Beyond demographic factors, BMI is also a significant predictor of depression risk in patients with DM. Utilizing Shapley Additive Explanations (SHAP) feature importance plots to quantify the contribution of each predictor to prediction outcomes, Sam et al. (27) identified BMI as a key factor in predicting depression risk in patients with DM, a finding consistent with the conclusions of Strine et al. (49). As a core clinical indicator for assessing obesity and overall nutritional status, abnormal BMI can influence metabolic homeostasis, physical activity, and psychological burden in patients with DM, thereby correlating with the onset and progression of depression risk (48, 50). Thus, BMI serves as an important link between physiological status and mental health. Beyond BMI, emerging evidence highlights the clinical significance of chest wall conformation, particularly convex-shaped chest morphology, which is associated with a higher comorbidity burden, diabetes prevalence, and depressive status. Recent findings in atrial fibrillation populations (51) suggest that specific thoracic anthropometrics may reflect systemic metabolic and inflammatory vulnerability. Future ML prediction models should focus on integrating these novel, easily assessable anthropometric markers into clinical routine. This not only enriches the theoretical framework of the biopsychosocial medical model but also enhances the feasibility and translational value of the prediction models in real clinical scenarios.

Marital status is another crucial predictor for depression comorbid with DM, with its mechanism of action closely tied to social support. Numerous domestic and international studies have indicated that patients with DM who are not married or lack family support face a higher risk of comorbid depression (52–54). This finding underscores the importance of social support for the mental health of this population. It supplements the influence of social factors beyond demographic and physiological indicators, rendering the prediction of depression risk more comprehensive.

Furthermore, the treatment modality for patients with DM is a key factor influencing their depression risk, with differential associations observed across various therapeutic regimens. A study focusing on elderly Chinese patients with T2DM found that the depression detection rate in the insulin therapy group was 35.0%, significantly higher than the 20.0% in the oral hypoglycemic drug group (55). Conversely, a study conducted in Serbia reported that patients using insulin exhibited milder depressive symptoms compared to those on oral hypoglycemic agents (56). Such contradictory results warrant further investigation. Mechanistically, patients receiving insulin therapy typically exhibit lower endogenous insulin levels and are more prone to metabolic dysregulation compared to those retaining some insulin secretory function, thereby increasing the risk of depression (57), which aligns with existing meta-analysis conclusions (58). Regarding oral hypoglycemic agents, some medications may cause adverse effects such as gastrointestinal discomfort and hypoglycemia (59), which can exacerbate psychological stress and indirectly elevate depression risk. Notably, however, metformin has been shown to alleviate depressive symptoms in patients with DM (60). The study by Chen et al. (61) further demonstrated that metformin use significantly reduces depression risk compared to untreated patients with DM, whereas other glucose-lowering agents lack similar effects. Additionally, psychological insulin resistance represents a non-negligible factor, defined as the reluctance exhibited by patients with DM and their physicians toward insulin therapy (62). Depression-related emotions arising from treatment, along with subsequent declines in treatment adherence and self-management behaviors, are considered key psychosocial pathways contributing to depression (62, 63).

These multifaceted findings spanning demographic, physiological, social, therapeutic, and psychological dimensions highlight the unique advantage of ML models in comprehensively capturing the multidimensional “bio-psycho-social” risks of depression. Clarifying these core predictors and their mechanisms not only provides a scientific basis for early screening of depression risk in patients with DM but also offers critical references for developing individualized prevention and intervention strategies in clinical settings, thereby facilitating comprehensive management of both physical and mental health in patients with DM.

### 4.4 Implications for future research and practice

In recent years, the development of predictive models based on artificial intelligence has become a focal point in medical research. In predicting depression risk among patients with DM, ML-based risk prediction models generally outperform traditional LR models. However, it is noteworthy that 6 studies (21, 22, 25, 29–31) demonstrated that LR exhibited superior predictive performance, suggesting that no modeling method holds an absolute advantage or disadvantage; the same approach may yield varying predictive capabilities across different research contexts and datasets. Consequently, when developing predictive models for depression risk in patients with DM, the indiscriminate pursuit of a singular methodology should be avoided. Instead, modeling strategies should be flexibly selected and optimized according to the specific research context and dataset characteristics. Furthermore, the regional subgroup analyses in this study revealed disparities in model performance across different populations. Additionally, most relevant studies rely on retrospective data, which further limits the external validity and generalizability of the models. Given these limitations, future efforts should prioritize conducting multicenter, multi-ethnic, and internationally collaborative prospective studies to enhance the applicability and clinical utility of models across diverse global populations. The exploration of novel, customized predictive indicators has the potential to overcome the performance bottlenecks of existing models and further improve predictive accuracy and the efficacy of individualized prevention (64). Epidemiological studies indicate that the development and progression of depression are closely associated with multidimensional factors, such as unhealthy lifestyles, dietary habits, emotional distress, and sleep disturbances (65). However, due to inconsistent evaluation standards and high subjectivity, these indicators are rarely included as candidate predictors in prediction models. Some studies have incorporated treatment-related variables, including treatment regimens, treatment burden, and medication status, which may enhance short-term predictive performance but compromise the model’s applicability in prospective clinical settings. It is recommended that future researchers fully adhere to the PROBAST-AI framework and, based on clinical practicality, select predictors that are easily measurable and amenable to intervention for model development. The impact of these factors on enhancing model performance should also be further investigated.

### 4.5 Limitations

This study has several limitations (1): Only English-language studies were included, potentially leading to publication bias owing to the exclusion of non-English studies (2); Some included studies failed to report expected/observed ratios or other metrics for assessing the calibration of risk prediction models, limiting the comprehensive assessment of their calibration performance (3); Most included studies adopted a cross-sectional design, which relies on single-timepoint data and fails to sufficiently establish causal relationships between predictors and outcomes, thereby constraining the predictive validity of the models (4); Although extensive subgroup analyses incorporating multiple factors were performed, they only partially accounted for heterogeneity without substantially mitigating its impact. Future research should further explore potential sources of heterogeneity while optimizing modeling approaches to enhance the interpretability and clinical utility of ML-based depression risk prediction models for patients with DM.

## 5 Conclusion

This systematic review evaluated 14 ML-based studies focusing on depression risk prediction models for patients with DM, with a comprehensive analysis of model performance, predictor selection, risk of bias, and clinical applicability. The findings demonstrated that the included models generally exhibited satisfactory predictive performance. However, the majority were developed using retrospective data and exhibited common limitations such as high risk of bias and insufficient external validation. Furthermore, methodological details and the imputation methods used to handle missing data were inadequately reported in several included studies, indicating the need for greater standardization. Future research should adhere to the TRIPOD reporting guidelines to refine study design and reporting, thereby enabling the development of robust, reproducible, and generalizable prediction models. Such models would assist clinical practitioners in accurately identifying patients with DM at high risk of depression and implementing timely preventive interventions, ultimately reducing individual medical costs and alleviating the financial burden on national healthcare systems.

## Data Availability

The original contributions presented in the study are included in the article/**Supplementary Material**. Further inquiries can be directed to the corresponding author.
