# Supplementary material for: Machine learning-based risk predictive models for depression in patients with diabetes: a systematic review and meta-analysis
Source: Front Endocrinol (Lausanne). 2026 Apr 15;17:1816661. doi: 10.3389/fendo.2026.1816661 (PMC13124507; doi:10.3389/fendo.2026.1816661)
Supplement: Supplementary file 1 [file DataSheet1.docx]

Supplementary Material

# Supplementary Figures and Tables

## Supplementary Figures


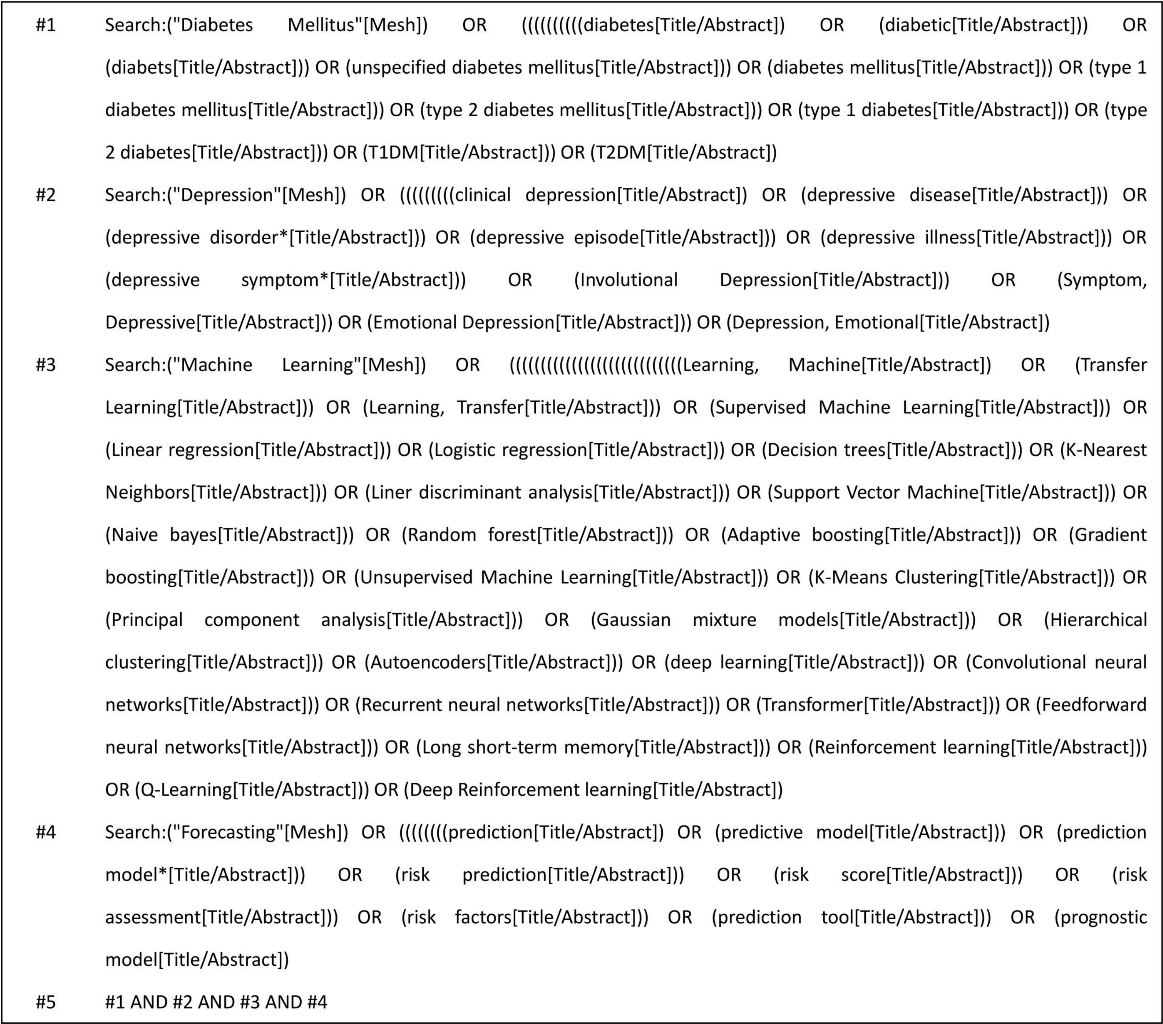


**Supplementary Figure 1** PubMed Search Strategy.


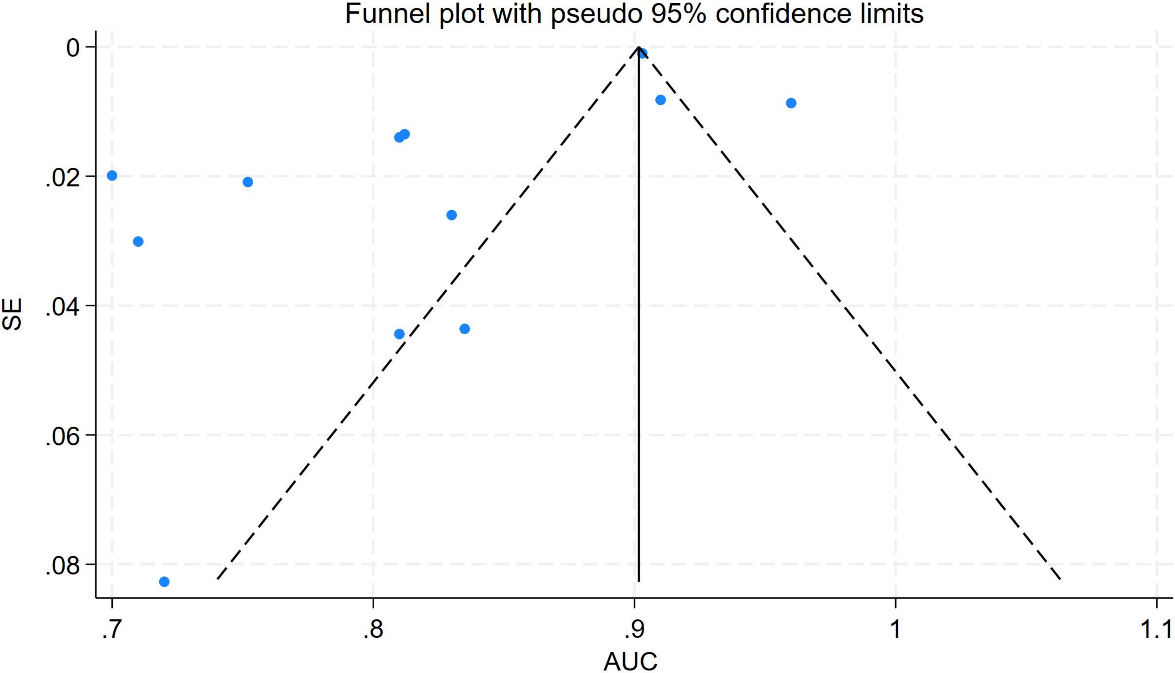


**Supplementary Figure 2** Funnel plot examination

**
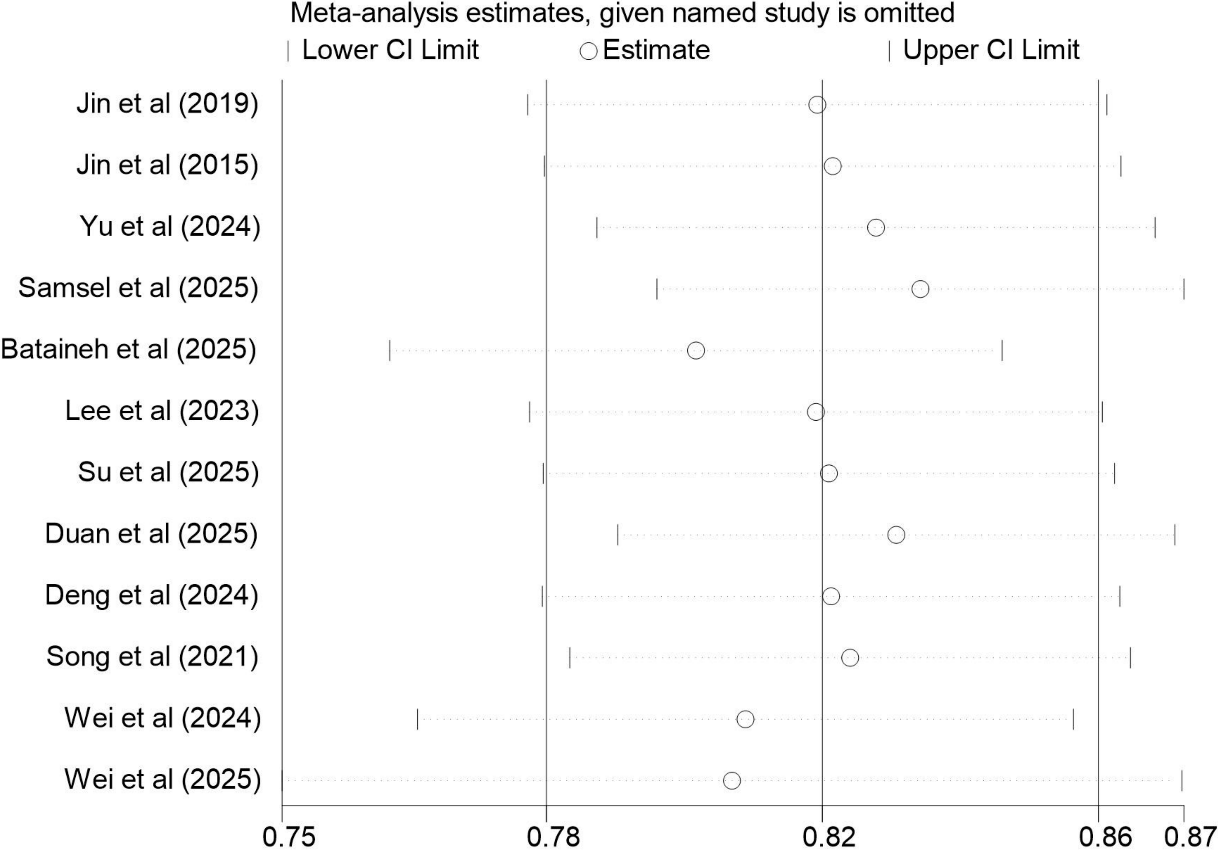
**

**Supplementary Figure 3** Sensitivity analysis


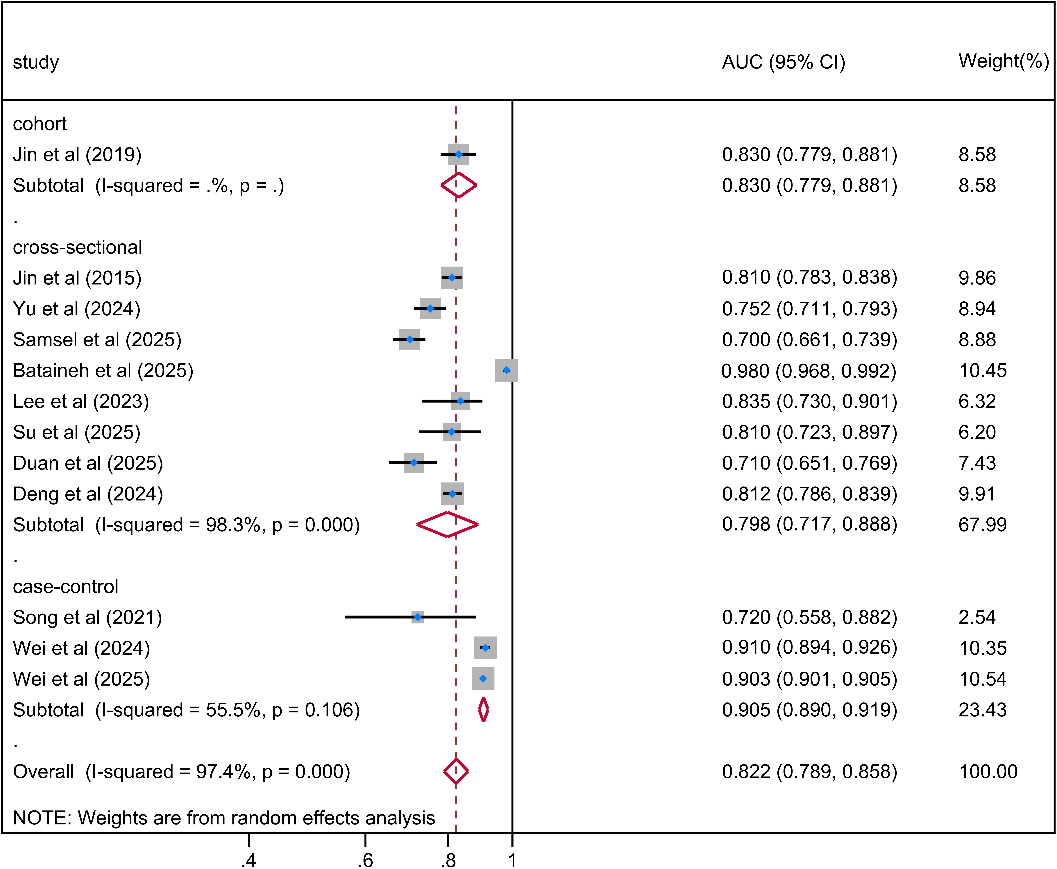


**Supplementary Figure 4** Random effects forest plot of AUC in study type subgroup for predicting depression risk in DM using ML.


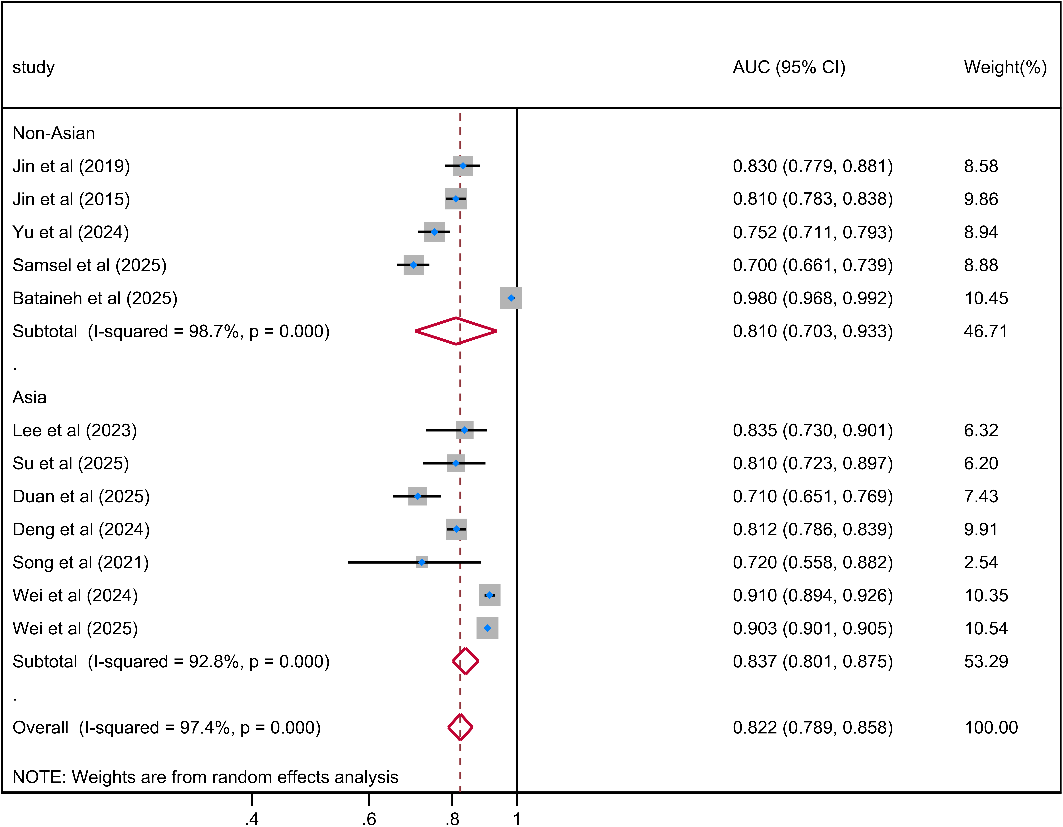


**Supplementary Figure 5** Random effects forest plot of AUC in country subgroup for predicting depression risk in DM using ML.

## Supplementary Tables

## Supplemental Table 1 Risk of bias for each study using PROBAST-AI Checklist

| **Study** | **Risk of bias assessment** | | | | **Applicability evaluation** | | | **Total** | |  |
| --- | --- | --- | --- | --- | --- | --- | --- | --- | --- | --- |
|  | **Participants** | **Predictors** | **Outcome** | **Analysis** | **Participants** | **Predictors** | **Outcome** | **Risk of bias** | **Applicability** | |
| Jin et al (2019) (21) | **＋** | **＋** | **－** | **－** | **＋** | **＋** | **＋** | **－** | **＋** | |
| Jin et al (2015) (22) | **－** | **－** | **＋** | **－** | **＋** | **＋** | **＋** | **－** | **＋** | |
| Khalil et al (2017) (23) | **－** | **－** | **－** | **－** | **＋** | **＋** | **＋** | **－** | **＋** | |
| Lee et al (2023) (24) | **－** | **－** | **＋** | **＋** | **＋** | **＋** | **＋** | **－** | **＋** | |
| Yu et al (2024) (25) | **－** | **－** | **＋** | **＋** | **＋** | **＋** | **＋** | **－** | **＋** | |
| Bourkhime et al (2025) (26) | **－** | **－** | **＋** | **－** | **＋** | **＋** | **＋** | **－** | **＋** | |
| Samsel et al (2025) (27) | **－** | **－** | **＋** | **－** | **＋** | **＋** | **＋** | **－** | **＋** | |
| Bataineh et al (2025) (28) | **－** | **－** | **－** | **－** | **＋** | **＋** | **＋** | **－** | **＋** | |
| Su et al (2025) (29) | **－** | **－** | **＋** | **－** | **＋** | **＋** | **＋** | **－** | **＋** | |
| Duan et al (2025) (30) | **－** | **－** | **＋** | **－** | **＋** | **＋** | **＋** | **－** | **＋** | |
| Deng et al (2024) (31) | **－** | **－** | **＋** | **＋** | **＋** | **＋** | **＋** | **－** | **＋** | |
| Song et al (2021) (32) | **＋** | **－** | **＋** | **－** | **＋** | **＋** | **＋** | **－** | **＋** | |
| Wei et al (2024) (33) | **－** | **－** | **＋** | **－** | **＋** | **＋** | **＋** | **－** | **＋** | |
| Wei et al (2025) (34) | **－** | **－** | **＋** | **－** | **＋** | **＋** | **＋** | **－** | **＋** | |

+, high risk of bias; **－**, low risk of bias
